# Supplementary material for: The impact of advance organizers in virtual classrooms on the development of integrated science process skills
Source: PeerJ Comput Sci. 2024 Apr 5;10:e1989. doi: 10.7717/peerj-cs.1989 (PMC11042017; doi:10.7717/peerj-cs.1989)
Supplement: Supplemental Information 2 [file peerj-cs-10-1989-s002.docx]

**Appendix A**

**Assessment Card of Integrated Science Process Skills**

| *Assessment Card Main Domains* | | *Strongly disagree* | *Disagree* | *Neutral* | *Agree* | *Strongly agree* |
| --- | --- | --- | --- | --- | --- | --- |
| 1. Procedural Definition | | | | | | |
| 1 | Considering criteria for research title's good formulation. |  |  |  |  |  |
| 2 | Writing the main question the research will address. |  |  |  |  |  |
| 3 | Identifying research population. |  |  |  |  |  |
| 4 | Formulating the phenomena and their conditions or terms associated with the result and its causes |  |  |  |  |  |
| 5 | Documenting resources and references. |  |  |  |  |  |
| 1. Variables Identification and Control | | | | | | |
| 6 | Identifying affecting variables. |  |  |  |  |  |
| 7 | Identifying persistent, independent and dependent variables. |  |  |  |  |  |
| 8 | Highlighting conditions for fixing and not fixing variables |  |  |  |  |  |
| 9 | Evaluation of the independent variable's impact on the dependent variable. |  |  |  |  |  |
| 10 | Controlling fixed variables. |  |  |  |  |  |
| 1. Questions and Hypotheses | | | | | | |
| 11 | Deciding the research questions to be answered. |  |  |  |  |  |
| 12 | Isolating direct questions from logical ones. |  |  |  |  |  |
| 13 | Splitting long or short questions |  |  |  |  |  |
| 14 | Formulating probable answers for every question. |  |  |  |  |  |
| 15 | Writing research alternative and null hypotheses |  |  |  |  |  |
| 16 | Recognizing hypotheses that are described quantitatively from those that can be tested descriptively. |  |  |  |  |  |
| 1. Procedures and Experimentation | | | | | | |
| 17 | Describing sampling type and technique |  |  |  |  |  |
| 18 | Defining proposed experimental design |  |  |  |  |  |
| 19 | Identifying the tools and techniques of data collection. |  |  |  |  |  |
| 20 | Following direct, clear, and simple steps. |  |  |  |  |  |
| 21 | Using appropriate equipment |  |  |  |  |  |
| 22 | Concluding results in a short time. |  |  |  |  |  |
| 1. Interpretation of Results | | | | | | |
| 23 | Identifying results or data relevant to the topic. |  |  |  |  |  |
| 24 | Processing results or data. |  |  |  |  |  |
| 25 | Identifying relevant principles, laws or theoretical frameworks. |  |  |  |  |  |
| 26 | Including answer of research in a prior research related to research chosen topic. |  |  |  |  |  |
